# Study protocol for a prospective, open-label, single-arm, phase II study on the combination of tislelizumab, nab-paclitaxel, gemcitabine, and concurrent radiotherapy as the induction therapy for patients with locally advanced and borderline resectable pancreatic cancer

**DOI:** 10.3389/fonc.2022.879661

**Published:** 2022-08-18

**Authors:** Changchang Lu, Yahui Zhu, Weiwei Kong, Ju Yang, Linxi Zhu, Lei Wang, Min Tang, Jun Chen, Qi Li, Jian He, Aimei Li, Xin Qiu, Qing Gu, Dongsheng Chen, Fanyan Meng, Baorui Liu, Yudong Qiu, Juan Du

**Affiliations:** ^1^ The Comprehensive Cancer Center of Drum Tower Hospital, Medical School of Nanjing University, Nanjing, China; ^2^ Nanjing Drum Tower Hospital Clinical College of Nanjing University of Chinese Medicine, Nanjing, China; ^3^ Department of Hepatopancreatobiliary Surgery, Drum Tower Hospital, Medical School of Nanjing University, Nanjing, China; ^4^ Digestive Department of Drum Tower Hospital, Medical School of Nanjing University, Nanjing, China; ^5^ Imaging Department of Drum Tower Hospital, Medical School of Nanjing University, Nanjing, China; ^6^ Pathology Department of Drum Tower Hospital, Medical School of Nanjing University, Nanjing, China; ^7^ Nuclear Medicine Department of Drum Tower Hospital, Medical School of Nanjing University, Nanjing, China; ^8^ National Institute of Healthcare Data Science at Nanjing University, Nanjing, China; ^9^ The State Key Laboratory of Translational Medicine and Innovative Drug Development, Medical Department, Jiangsu Simcere Diagnostics Co., Ltd, Nanjing, China

**Keywords:** PD-1 blockade, induction therapy, circulating tumor DNA, clinical protocol, pancreatic cancer

## Abstract

**Background:**

Pancreatic ductal adenocarcinoma (PDAC) is a fatal malignancy with a low resection rate. Chemotherapy and radiotherapy (RT) are the main treatment approaches for patients with advanced pancreatic cancer, and neoadjuvant chemoradiotherapy is considered a promising strategy to increase the resection rate. Recently, immune checkpoint inhibitor (ICI) therapy has shown remarkable efficacy in several cancers. Therefore, the combination of ICI, chemotherapy, and concurrent radiotherapy is promising for patients with potentially resectable pancreatic cancer, mainly referring to locally advanced (LAPC) and borderline resectable pancreatic cancer (BRPC), to increase the chances of conversion to surgical resectability and prolong survival. This study aims to introduce the design of a clinical trial.

**Methods:**

This is an open-label, single-arm, and single-center phase II trial. Patients with pathologically and radiographically confirmed LAPC or BRPC without prior anti-cancer treatment or severe morbidities will be enrolled. All patients will receive induction therapy and will be further evaluated by the Multiple Disciplinary Team (MDT) for the possibility of surgery. The induction therapy consists of up to four cycles of gemcitabine 1,000 mg/m^2^ and nab-paclitaxel 125 mg/m^2^ *via* intravenous (IV) infusion on days 1 and 8, along with tislelizumab (a PD-1 monoclonal antibody) 200 mg administered through IV infusion on day 1 every 3 weeks, concurrently with stereotactic body radiation therapy (SBRT) during the third cycle of treatment. After surgery, patients without progression will receive another two to four cycles of adjuvant therapy with gemcitabine, nab-paclitaxel, and tislelizumab. The primary objectives are objective response rate (ORR) and the R0 resection rate. The secondary objectives are median overall survival (mOS), median progression free survival (mPFS), disease control rate (DCR), pathological grade of tumor tissue after therapy, and adverse reactions. Besides, we expect to explore the value of circulating tumor DNA (ctDNA) in predicting tumor response to induction therapy and survival outcome of patients.

**Discussion:**

This is a protocol for a clinical trial that attempts to evaluate the safety and efficacy of the combination of anti-PD-1 antibody plus chemotherapy and radiotherapy as the induction therapy for LAPC and BRPC. The results of this phase II study will provide evidence for the clinical practice of this modality.

**Clinical Trial Registration:**

http://www.chictr.org.cn/edit.aspx?pid=53720&htm=4, identifier ChiCTR2000032955.

## Background

Pancreatic ductal adenocarcinoma (PDAC) is a highly fatal disease with increasing incidence rates. It is predicted to become the third leading cause of cancer-related death in the United States by 2030 (1). The major reasons include limited effective therapeutic options and severe mortality rates. The standard treatment is surgery followed by adjuvant chemotherapy (2). However, only approximately 15%–20% of all patients are deemed resectable, and many of them are potentially resectable (30%–40%), including those with locally advanced pancreatic cancer (LAPC) and borderline resectable pancreatic cancer (BRPC). Furthermore, approximately 50% of patients are diagnosed with distant metastasis (3–5). Additionally, the 5-year overall survival (OS) rate remains in the single digits despite advances in medicine and surgical techniques (6, 7).

The purpose of adjuvant therapy is to reduce postsurgical recurrence and prolong survival (2). However, the main aim of neoadjuvant therapy is to improve the patient selection for surgical intervention and increase the potential for R0 resection (8). A meta-analysis of FOLFIRINOX as a first-line treatment for LAPC analyzed 13 studies and revealed that the median OS is 24.2 months and the median progression free-survival (PFS) is 15 months (9). Another meta-analysis of 14 studies involving 365 patients with LAPC concluded that the median OS is 8.9–25.0 months and the resection rate is 28% (10). Additionally, the median OS of patients with BRPC who received neoadjuvant therapy such as FOLFIRINOX and gemcitabine-based chemoradiotherapy was approximately 17–22.2 months and the R0 resection rate of patients in the immediate surgery group was almost half that of patients treated with neoadjuvant therapy (20%–40% vs 50%–80%) (11–13). A study demonstrated that the median OS for patients with BRPC and LAPC who underwent resection after neoadjuvant therapy was 37.7 months (14). The currently used neoadjuvant regimens for PDAC are FOLFIRINOX (5-fluorouracil, oxaliplatin, irinotecan, and leucovorin) and gemcitabine and/or nab-paclitaxel (15). Besides chemotherapy, radiotherapy (RT) is included in neoadjuvant therapy, especially stereotactic body radiotherapy (SBRT), which has been proven to be safe and effective (16).

Recently, immune checkpoint inhibitor (ICI) with PD-1/PD-L1 antibodies has displayed remarkable efficacy in several cancers, particularly lung cancer, melanoma, and renal cancer (17). Nevertheless, the treatment of PDAC with a single PD-1 inhibitor was not effective since most patients had a low tumor mutational burden (TMB-L) (18). Some reports have shown that the anti-PD-1 antibodies applied to neoadjuvant chemoimmunotherapy exhibit promising effects for treating gastric cancer (19), esophageal squamous cell carcinoma (20), and non-small cell lung cancer (21). Tislelizumab is one of the humanized IgG4 anti-PD-1 monoclonal antibody which has shown potential against gastrointestinal cancers such as esophageal cancer (22), gastric cancer (23), biliary tract cancer (24), and so on. We conducted a phase II study enrolling 50 patients with LAPC or BRPC to evaluate the safety and efficacy of this regimen (tislelizumab plus gemcitabine and nab-paclitaxel (AG) and sequential SBRT).

Circulating tumor DNA (ctDNA) containing tumor-specific DNA mutations can be detected in the cell-free component of peripheral blood in most patients with PDAC (25). For resectable pancreatic cancer, post-surgical ctDNA detection was an independent negative predictor of decreased recurrence-free survival and OS (26). As for localized pancreatic cancer, detectable ctDNA post-operatively appeared to have a higher risk of recurrence on gemcitabine-based adjuvant therapy (27). For early cancer detection, ctDNA assays hold substantial potential as a cancer screening test (28). However, whether the genomic features and the serial ctDNA status can predict outcomes of patients in the context of chemoimmunotherapy is still under investigation. In this study, we aim to investigate the role of genomic mutation features and serial ctDNA dynamic change in predicting tumor response and outcomes in patients with LAPC and BRPC. This trial is expected to demonstrate the feasibility of the combined therapeutic approach as the induction treatment in patients with LAPC and BRPC and provide evidence for further research on the same.

## Method

### Trial design

**Figure 1:**
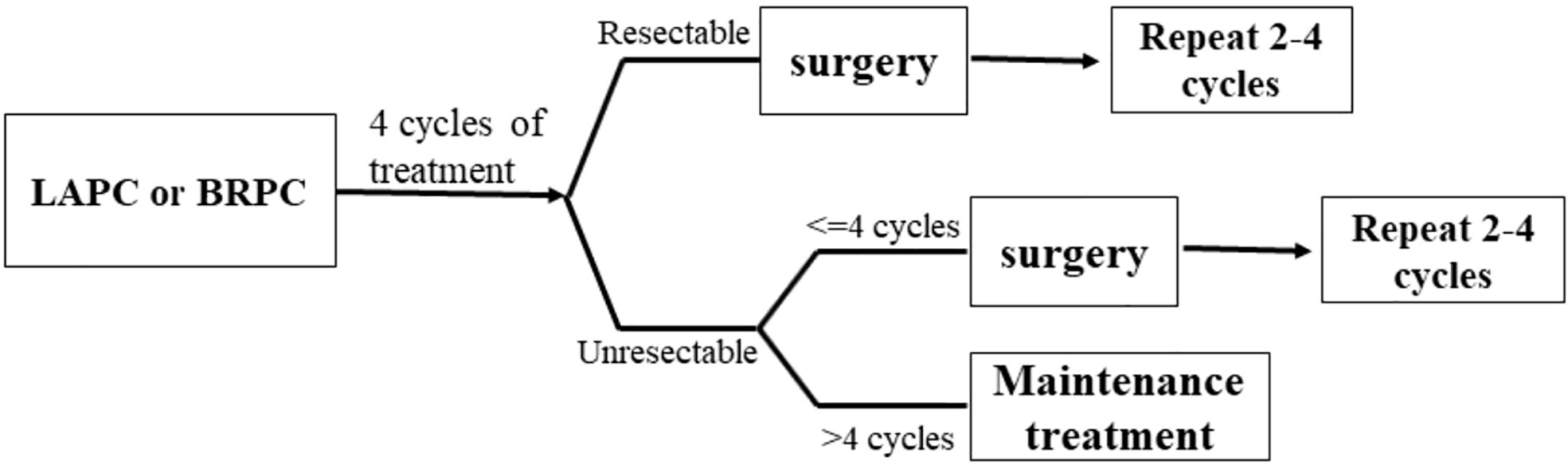
The process of the clinical trial.

**Figure 2:**
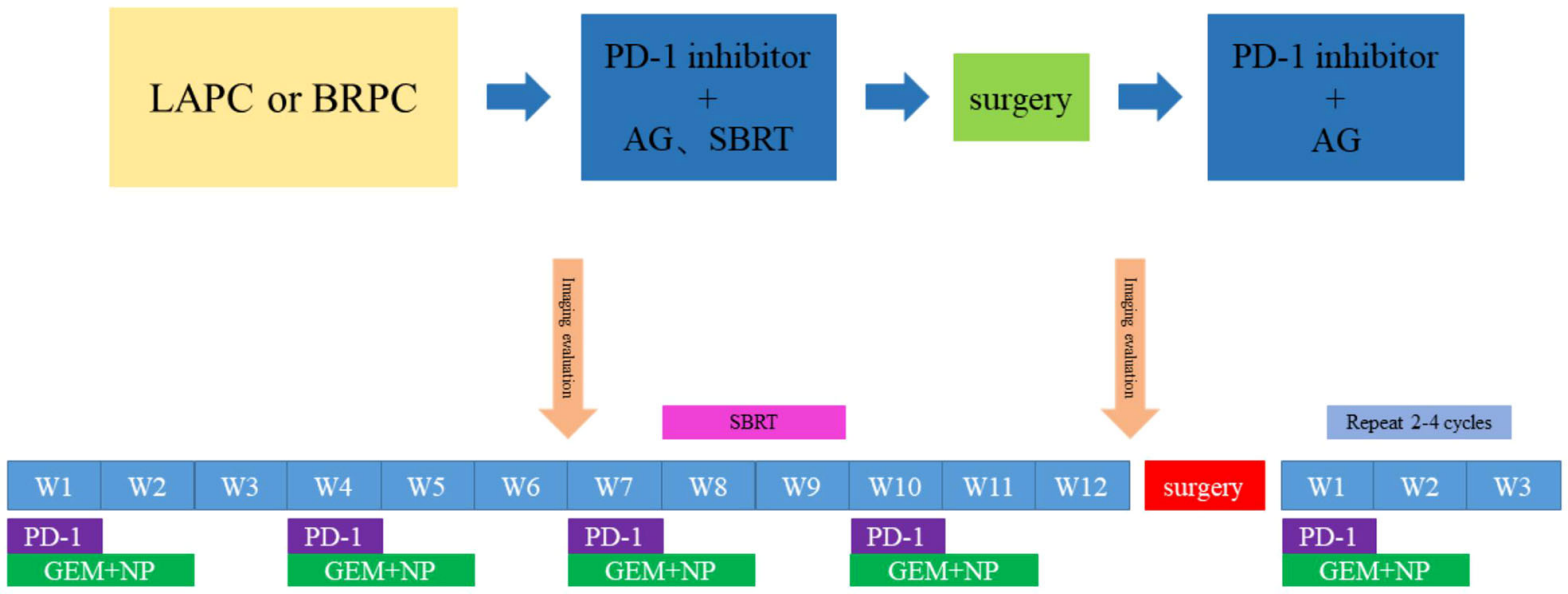
Timeline of the treatment. Every 3 weeks one cycle: Tislelizumab, (200 mg, on day 1), gemcitabine (1,000 mg/m^2^, on days 1 and 8) and nab-paclitaxel (125 mg/m^2^, on days 1 and 8); Radiotherapy: PTV:30Gy/10f; PGTV:50Gy/10f.

This is a prospective, open-label, single-arm, and single-center phase II study. We enrolled patients aged ≥18 years with histologically or cytologically and radiographically confirmed LAPC or BRPC. We classified LAPC and BRPC according to the National Comprehensive Cancer Network Guidelines, as indicated in **Table 1**. Details of inclusion and exclusion criteria are presented in **Table 2**. The regimen consists of four cycles of gemcitabine 1,000 mg/m^2^ and nab-paclitaxel 125 mg/m^2^ administered on days 1 and 8 *via* intravenous (IV) infusion, along with tislelizumab 200 mg administered *via* IV infusion on day 1 every 3 weeks. Concurrent chemoradiotherapy is administered after two cycles of systemic therapy. Patients will undergo CT simulation for radiation treatment planning about one week after two cycles of chemotherapy plus immunotherapy. We create a gross target volume (GTV) following the complete extent of the tumor delineated in each CT phase, and a clinical target volume (CTV) is an expansion of 0.5 cm around the GTV, including lymph node area. The planning organ at risk volume (PRV) is a 1-cm expansion of the gastrointestinal tracts. We generate planned gross tumor volume (PGTV) as a 0.3-cm expansion from GTV and subtract PRV. The planning target volume (PTV) encompasses a 0.5-cm expansion of CTV. Patients will be administered SBRT every day with a total dose of 30 Gy/10 f at PTV and 50 Gy/10 f at PGTV, simultaneously with the third cycle of chemotherapy and immunotherapy, about one week after CT simulation. Patients with disease progression (PD), including local progression, will change drugs and withdraw from the trial. For subsequent treatment, radiotherapy may be added to the second-line treatment. However, the patients will not continue the clinical trial. An imaging evaluation will be performed every 6 weeks. When induction treatment is completed, surgery will be decided by the Multiple Disciplinary Team (MDT). The MDT consists of multidisciplinary experts in pancreatic cancer, including medical oncologists, pancreatic surgeons, gastroenterologists, radiologists, pathologists, imaging specialists, and a coordinator. Patients without PD will continue to receive two to four cycles of adjuvant treatment with the same systemic regimen as in the induction phase. However, if the MDT team believes that the tumor remains unresectable, the patient will continue the existing treatment until there are indications for surgery. In addition, patients who do not undergo surgery after eight cycles of treatment will receive maintenance treatment, which consists of gemcitabine 1,000 mg/m^2^, nab-paclitaxel 125 mg/m^2^, and tislelizumab 200 mg once a month. The main process of this trial and the treatment schedule are demonstrated in **Figures 1**, **2**. Written informed consent will be obtained from all patients before enrollment. In this study, treatment continued till there was the presence of PD, intolerable toxicity, withdrawal of consent, or any other reason to discontinue it. For grade 3 or higher adverse events (AEs), the treatment should be suspended and the AEs will be treated until they returned to normal or grade 1 or 2. The treatment dose will be reduced as decided by the researchers. In addition, the medical safety team will monitor all the safety information during this study. Patients with early termination during the study will receive an alternative treatment of the choice of the investigator. The study was performed as per the Declaration of Helsinki. The protocol was reviewed and approved by the Medical Ethics Committee of Drum Tower Hospital affiliated with Nanjing University Medical School.

**Table 1:** Definition of locally advanced and borderline resectable pancreatic cancer from the NCCN-Guidelines (version 1.2021).

Vessel involvement	Borderline resectable	Locally advanced	
**Arterial vessels**	**Pancreatic head/uncinate process:** 1. Contact with CHA without extension to CA or hepatic artery bifurcation allowing for safe and reconstruction. 2. Contact with the SMA of ≤180°. 3. Contact with variant arterial anatomy (ex: accessory right hepatic artery, replaced right hepatic artery, replaced CHA, and the origin of replaced or accessory artery) and the presence and degree of tumor contact should be noted if present, as it may affect surgical planning.	**Pancreatic head/uncinate process:** 1. Contact with SMA >180°. 2. Contact with the CA >180°. Pancreatic body/tail: 1. Contact of >180° with the SMA or CA. 2. Contact of the CA and aortic involvement.	
	**Pancreatic body/tail:** 1. Contact with the CA of ≤180°. 2. Contact with the CA of >180° without involvement of the aorta and with intact and uninvolved gastroduodenal artery thereby permitting a modified Appleby procedure.		
**Venous vessels**	1. Contact with the SMV or PV of >180°, contact of ≤180° with contour irregularity of the vein or thrombosis of the vein but with suitable vessel proximal and distal to the site of involvement allowing for safe and complete resection and vein reconstruction. 2. Contact with the inferior vena cava (IVC).	Unreconstructible SMV/PV due to tumor involvement or occlusion (can be due to tumor or bland thrombus)	

CHA, common hepatic artery; SMA, superior mesenteric artery; CA, celiac axis; SMV, superior mesenteric vein; PV, portal vein.

**Table 2:** The inclusion and exclusion criteria of the protocol.

Inclusion criteria	Exclusion criteria
a. Subjects with age ≥18 years and ECOG score of 0–1;	a. Patients who have received systematic anti-tumor treatment.
b. Subjects with pancreatic cancer confirmed by histology or cytology;	b. Patients with previous history of other tumors, except for cervical cancer in situ, treated squamous cell carcinoma or bladder epithelial tumor (TA and TIS) or other malignant tumors that have received radical treatment (at least 5 years before enrollment).
c. The patients with potentially resectable pancreatic cancer were imaged;	c. Patients with active bacterial or fungal infection (≥level 2 of NCI-CTC, 3rd Edition).
d. The subjects should meet the following hematological indexes: Neutrophil count ≥1.5 ∗ 10^9^/L, Hemoglobin ≥10 g/dl Platelet count ≥100 ∗ 10^9^/L;	d. Patients with HIV, HCV, HBV infection, uncontrollable coronary artery disease or asthma, uncontrollable cerebrovascular disease or other diseases considered by researchers to be out of the group.
e. The subjects should meet the following biochemical indicators: Total bilirubin ≤1.5 ∗ULN; AST and ALT <1.5 ∗ ULN; Creatinine clearance rate ≥60 ml/min;	e. Patients with autoimmune diseases or immune defects who are treated with immunosuppressive drugs.
f. Subjects of childbearing age need to take appropriate protective measures (contraceptive measures or other methods of birth control) before entering the group and during the test.	f. Pregnant and lactating women. Pregnant women of childbearing age must be tested negative within 7 days before entering the group.
g. Subjects who have signed informed consent;	g. Patients with drug abuse, clinical or psychological or social factors make informed consent or research implementation affected.
h. Subjects who were able to follow the protocol and follow-up procedures.	h. Patients who may be allergic to PD-1 monoclonal antibody immunotherapy drugs.

ECOG, Eastern Cooperative Oncology Group ULN, Upper Limit of Normal; ILD, interstitial lung disease.

### Research hypothesis

The combination therapy of tislelizumab plus AG and concurrent radiotherapy as the induction therapy improves the ORR and R0 resection rate and prolongs the PFS and OS for patients with locally advanced and borderline resectable pancreatic cancer with an acceptable safety profile.

### Objectives

#### Primary objectives

The primary objectives of the study included evaluating ORR and R0 resection rates in the intent-to-treat (ITT) population. ITT refers to patients who complete the treatment as planned. ORR is defined as the proportion of patients with the best overall response of complete response (CR) or partial response (PR) according to the Response Evaluation Criteria in Solid Tumors (RECIST). R0 resection is defined as no tumor cells visualized at the surgical margin under the microscope.

#### Secondary objectives

The secondary objectives of the study included evaluating OS and PFS in the ITT population. Disease control rate (DCR), pathological response rate, and adverse reaction rate are included. OS is defined as the time from the date of randomization to the date of death due to any cause. PFS is defined as survival without any progressive disease and distant metastases from the date of randomization. DCR refers to the number of cases with remission and stable disease accounting for the ITT population. Safety evaluation includes the tolerance of patients to this regimen and the influence of these drugs on the skin, digestive tract, and electrolyte balance of patients. The reasons for dropouts include PD, death, adverse reactions, and the wishes of the patient. For patients with surgical resection, pathological response is one of the observed objectives.

#### Exploratory objectives

The exploratory objectives of this trial are to further investigate the predictive biomarkers for the efficacy of the combination therapy. Genomic mutation features and the serial ctDNA status *via* next-generation sequencing will be investigated in the induction therapy. Peripheral blood samples will be collected from each patient in ethylene diamine tetra acetic acid (EDTA) vacutainer tubes at four time points, i.e., before induction therapy (baseline), two cycles after the initiation of induction therapy, after radiation treatment or before surgery (preop), and within 1 month after surgery (postop) or at the time of progression. All the extraction procedures will be performed following the instructions of the manufacturer. DNA was quantified on a Qubit Fluorometer with a Qubit dsDNA HS Assay Kit (Thermo) and its quality was evaluated by the Agilent 4200 TapeStation (Agilent).

### The course of the trial

The main process of the trial is summarized in **Figure 1**. Every patient is confirmed to have a diagnosis through imaging and pathological evidence, such as computed tomography (CT) and endoscopic ultrasound-guided-fine-needle aspiration (EUS-FNA) before enrollment. The patient needs to spend at least 8 months completing the planned treatment. All adverse effects that occur during the treatment are recorded and corresponding measures will be taken. Patients will discontinue the treatment if they have PD or intolerable toxicities. However, we will provide them with optimal second-line therapy and continue follow-up until death.

For patients with PR after finishing at least four cycles of SBRT, the feasibility of surgical resection will be discussed by MDT. The patients who successfully receive resection need to recuperate for almost one month and are further evaluated using imaging for therapeutic efficacy. If the patient recovers well, we will treat them with another two or more cycles of adjuvant therapy.

The course of the trial mainly consists of four phases: screening, baseline evaluation, treatment, and follow-up.

### Screening

The screening of patients will be accomplished within one week before the initial treatment. Patients are screened mainly according to the inclusion and exclusion criteria. We record medical history, family history, and ECOG score and conduct physical examinations, including weight, height, etc. Patients also undergo blood tests to detect liver and kidney function. Before enrollment, the examination results of patients will be re-assessed to assess their eligibility. All treatments are provided after receiving informed consent from the patients.

### Baseline evaluation

The baseline evaluation of the patient will be completed within two weeks of the treatment. It mainly includes medical history, physical examination, hematological examination, and other laboratory tests (such as CEA, CA199, and CA125), pathological examination, CT or MRI scan, and ECOG score. Positron emission tomography/computed tomography (PET-CT) and gene detection are optional for the enrolled patients.

### Treatment

According to the treatment schedule, nab-paclitaxel 125 mg/m^2^ and gemcitabine 1,000 mg/m^2^ will be administered through IV on days 1 and 8, and tislelizumab will be administered through IV as 200 mg on day 1, and will be repeated every 21 days. Protocol-specified treatment modifications will be undertaken when severe toxicity occurs, and all the side effects will be recorded. Patients with no progression after two cycles will receive concurrent radiotherapy in the third cycle. The planned dose of SBRT is 30 Gy/10 f at PTV and 50 Gy/10 f at PGTV. When four cycles of medication and radiotherapy are completed, surgeries will be carried out as discussed by the MDT. All patients who undergo pancreatectomy must receive another two or more cycles of adjuvant therapy. Patients with PD during the treatment will be evaluated for possible second-line therapy. All combined treatments should be recorded in the case report form (CRF).

### Follow-up

For patients who complete the scheduled treatment, including induction and adjuvant therapy, follow-up will start one month after the end of the treatment. Furthermore, we will record survival and progression status *via* face-to-face visits or phone calls every two or three months. For patients with PD during treatment, we will still perform their follow-up every two or three months and record the latest treatment and survival.

### Outcome measures

#### Clinical efficacy assessment

Comprehensive information about eligible patients will be collected before the initiation of the treatment according to baseline assessment. AEs and severe adverse events (SAEs) will be evaluated according to NCI-CTCAE v5.0 during the therapy. Patients are subjected to imaging evaluation every two cycles. After four cycles of chemoimmunotherapy and completion of radiotherapy, MDT will comprehensively evaluate whether the surgery can be performed based on the tumor size and its relationship with surrounding vessels on CT scan and the change in tumor markers. Exploration for resection is considered if there is a decrease >50% in the CA19-9 level according to the National Comprehensive Cancer Network (NCCN) Guideline. We will also compare the standard uptake value (SUV) and observe no distant metastasis through PET/CT. Additionally, the postsurgical pathology of patients will be graded according to the American Joint Committee on Cancer (AJCC). The patients are scheduled for re-examination almost one month after the surgery and further receive at least two cycles of adjuvant treatment.

### Pharmacodynamic analysis

To explore the value of ctDNA as a tumor biomarker in clinical settings, peripheral blood samples of patients are collected to detect ctDNA in four periods, including before treatment, after two cycles, after radiotherapy, and after surgery. We will screen out several hot spot genes through ctDNA detection and evaluate the roles of these gene mutations in different treatment stages. Finally, we will use the Cox proportional hazard regression analysis to investigate the relationship between tumor response and gene mutations.

### Safety assessment

Adverse events will be summarized and graded according to general standards. Laboratory parameters will be reported with descriptive statistics. We will observe and record all AEs (including SAEs, radiotherapy-related adverse events, and immune-related adverse events (irAEs)). In this study, the following AEs are mainly considered: hematological and non-hematological toxicity, postoperative complications, death, and life-threatening AEs. Once the above adverse events occur, we will take relevant treatment measures, including dose or drug adjustment, and in severe cases, the current treatment will be temporarily stopped and symptomatic treatment will be given first.

### Statistical considerations

#### Estimated number of enrollments

This is an open-label, single-arm, phase II clinical trial and a planned 25 eligible subjects will be enrolled. There was no statistical hypothesis to determine the sample size. All demographic and clinical characteristics recorded at baseline will be displayed using descriptive analyses employing lists and tables. Observed rates and the exact binomial confidence interval (CI) of 95% will be calculated for ORR when the induction phase is completed. After a phase is accomplished, time-to-event variables, such as PFS and OS, will be estimated using the Kaplan–Meier method and plotted over time. Median PFS and OS will be presented along with their 95% CI, if estimable. For patients who undergo surgery, the pathological response rate and the R0 resection rate will be recorded and calculated. The T-test, Fisher’s test, and other appropriate tests will be used wherever applicable. The level of significance is P <0.05.

## Discussion

This trial aims to explore the efficacy and safety of the combination of a PD-1 inhibitor plus chemotherapy and radiotherapy as the induction therapy for patients with potentially resectable PDAC. Neoadjuvant therapy is a promising strategy to increase opportunities for undergoing surgery and an R0 resection rate for patients with potentially resectable pancreatic cancer. One of the largest meta-analyses of 111 studies (n = 4,394), including 56 phase I-II trials from 1966 to 2009, revealed that almost one-third of initial patients with unresectable PDAC may require surgery after neoadjuvant therapy (29). A small clinical trial demonstrated that the R0 resection rate was 80% after neoadjuvant chemotherapy for patients with resectable PDAC (30). In addition, a phase II clinical trial of neoadjuvant therapy with FOLFIRINOX, losartan, and radiotherapy for LAPC observed that R0 resection was achieved in 34 of 49 patients (69%) (31). We selected gemcitabine and nab-paclitaxel as the chemotherapy regimen, which has exhibited remarkable effects for LAPC in the LAPACT study (32). Compared with presurgical chemotherapy alone, chemoradiotherapy for LPAC or BRPC revealed a higher R0 resection rate and a lower rate of pathologic lymph node positivity (13, 33). For most patients with potentially resectable PDAC, surgical resection was previously not considered an option secondary to the invasion of adjacent vessels (34). Radiotherapy can induce vascular normalization to a certain extent (35) and is beneficial for local control (36). Besides, induction therapy helps prolong the survival of patients. A study analyzed the OS of 409 patients with localized PDAC treated with different therapies, and the result revealed that patients who underwent resection after induction therapy had a significantly longer survival time than those who had surgery directly (37).

It is widely accepted that PDAC is immunotherapy-resistant and checkpoint inhibitor monotherapy is limited to a few patients with PDAC mismatch repair (MMR) deficiency (38). The dense extracellular matrix of the tumor blocks the infiltration of immune cells and suppresses the immune response (39). However, the combination of chemotherapeutics and PD-1 blockade can enhance the Th1 response and promote the anticancer effect (40). Moreover, several animal and human studies have revealed that radiotherapy enhances the effect of immunotherapy by accentuating the uptake of tumor antigens and the migration of the activated effector T cells to the tumor (41). Additionally, it is noteworthy that ablative RT is more effective than fractionated RT in recruiting T cells. The RT-disturbed tumor microenvironment presented a higher vessel perfusion rate. The increase in vessel perfusion promotes the delivery of a higher number of anti-PD-L1 antibodies against the tumor. Hence, several researchers have supported the combination of PD-1 blockade and neoadjuvant chemoradiotherapy for treating gastrointestinal cancer (42, 43).

Additionally, we expect to explore more clinical biomarkers that can predict tumor response to induction therapy, monitor tumor burden, and estimate the prognosis for survival after treatment. Currently, the only prognostic biomarker for PDAC approved by the US Food and Drug Administration is CA19-9, which is low positive in patients who are asymptomatic or Lewis-negative (36, 44). Genomic features are believed to hold great potential to predict tumor response to cancer therapy, and a large sample analysis has demonstrated that ctDNA may be a feasible biomarker for various solid tumor types with clinical indications (45). Besides, some reports have suggested that ctDNA has the potential value of predicting the effects of immunotherapy in patients, and is an accurate dynamic biomarker to reflect the real-time tumor bulk (46–48). This is a prospective clinical trial that adopts a new regimen of induction therapy for patients with potentially resectable pancreatic cancer, and we expect that the result of the trial will demonstrate that the combination of PD-1 inhibitors and neoadjuvant chemoradiotherapy is effective and safe for patients with potentially resectable PDAC.

## Data availability statement

The original contributions presented in the study are included in the article/supplementary material. Further inquiries can be directed to the corresponding authors.

## Author contributions

CL and YZ have contributed equally to this work. BL, YQ, and JD are responsible for the conception of the trial. WK, LW, MT, JC, QL, JH, and AL were involved in the design of the trial. CL, YZ, XQ, QG, and LZ were involved in the acquisition and analysis of data for the trial. CL and YZ were involved in drafting the manuscript. JY, FM, and DC were involved in revising the manuscript. All authors have agreed the final version of manuscript.

## Funding

The study was supported by the National Key Research and Development Program of China (2020YFA0713804), the National Natural Science Foundation of China (82072926), the Special Fund of Health Science and Technology Development of Nanjing (YKK20080), and the Beijing Medical Award Foundation (YXJL-2020-0236-0050).

## Acknowledgments

We thank all the investigators who have contributed to this study, and we are grateful for the understanding and cooperation of patients.

## Conflict of interest

Author DC was employed by Jiangsu Simcere Diagnostics Co., Ltd.

The remaining authors declare that the research was conducted in the absence of any commercial or financial relationships that could be construed as a potential conflict of interest.

## Publisher’s note

All claims expressed in this article are solely those of the authors and do not necessarily represent those of their affiliated organizations, or those of the publisher, the editors and the reviewers. Any product that may be evaluated in this article, or claim that may be made by its manufacturer, is not guaranteed or endorsed by the publisher.

## References

[1] Rahib L Smith BD Aizenberg R Rosenzweig AB Fleshman JM Matrisian LM. Projecting cancer incidence and deaths to 2030: The unexpected burden of thyroid, liver, and pancreas cancers in the united states. Cancer Res (2014) 74:2913–21. doi: 10.1158/0008-5472.CAN-14-0155 24840647

[2] Heinrich S Lang H. Neoadjuvant therapy of pancreatic cancer: Definitions and benefits. Int J Mol Sci (2017) 18:1622–37. doi: 10.3390/ijms18081622 PMC5578014 28933761

[3] Klaiber U Hackert T. Conversion surgery for pancreatic cancer-the impact of neoadjuvant treatment. Front Oncol (2019) 9:1501. doi: 10.3389/fonc.2019.01501 31993372 PMC6971165

[4] Bockhorn M Uzunoglu FG Adham M Imrie C Milicevic M Sandberg AA. Borderline resectable pancreatic cancer: A consensus statement by the international study group of pancreatic surgery (ISGPS). Surgery (2014) 155:977–88. doi: 10.1016/j.surg.2014.02.001 24856119

[5] Kim HS Jang JY Han Y Lee KB Joo I Lee DH. Survival outcome and prognostic factors of neoadjuvant treatment followed by resection for borderline resectable pancreatic cancer. Ann Surg Treat Res (2017) 93:186–94. doi: 10.4174/astr.2017.93.4.186 PMC5658300 29094028

[6] Sun H Ma H Hong G Sun H Wang J. Survival improvement in patients with pancreatic cancer by decade: A period analysis of the SEER database, 1981-2010. Sci Rep (2014) 4:6747. doi: 10.1038/srep06747 25339498 PMC5381379

[7] Rangelova E Wefer A Persson S Valente R Tanaka K Orsini N. Surgery improves survival after neoadjuvant therapy for borderline and locally advanced pancreatic cancer: A single institution experience. Ann Surg (2021) 273:579–86. doi: 10.1097/SLA.0000000000003301 30946073

[8] di Sebastiano P Grottola T di Mola FF. Borderline resectable pancreatic cancer and the role of neoadjuvant chemoradiotherapy. Updates Surg (2016) 68:235–9. doi: 10.1007/s13304-016-0392-x 27629483

[9] Suker M Beumer BR Sadot E Marthey L Faris JE Mellon EA. FOLFIRINOX for locally advanced pancreatic cancer: A systematic review and patient-level meta-analysis. Lancet Oncol (2016) 17:801–10. doi: 10.1016/s1470-2045(16)00172-8 PMC5527756 27160474

[10] Rombouts SJ Walma MS Vogel JA van Rijssen LB Wilmink JW Mohammad NH. Systematic review of resection rates and clinical outcomes after FOLFIRINOX-based treatment in patients with locally advanced pancreatic cancer. Ann Surg Oncol (2016) 23:4352–60. doi: 10.1245/s10434-016-5373-2 PMC5090009 27370653

[11] Janssen QP Buettner S Suker M Beumer BR Addeo P Bachellier P. Neoadjuvant FOLFIRINOX in patients with borderline resectable pancreatic cancer: A systematic review and patient-level meta-analysis. J Natl Cancer Inst (2019) 111:782–94. doi: 10.1093/jnci/djz073 PMC6695305 31086963

[12] Janssen QP O'Reilly EM van Eijck CHJ Groot Koerkamp B. Neoadjuvant treatment in patients with resectable and borderline resectable pancreatic cancer. Front Oncol (2020) 10:41. doi: 10.3389/fonc.2020.00041 32083002 PMC7005204

[13] Versteijne E Suker M Groothuis K Akkermans-Vogelaar JM Besselink MG Bonsing BA. Preoperative chemoradiotherapy versus immediate surgery for resectable and borderline resectable pancreatic cancer: Results of the Dutch randomized phase III PREOPANC trial. J Clin Oncol (2020) 38:1763–73. doi: 10.1200/JCO.19.02274 PMC8265386 32105518

[14] Michelakos T Pergolini I Castillo CF Honselmann KC Cai L Deshpande V. Predictors of resectability and survival in patients with borderline and locally advanced pancreatic cancer who underwent neoadjuvant treatment with FOLFIRINOX. Ann Surg (2019) 269:733–40. doi: 10.1097/SLA.0000000000002600 29227344

[15] Raufi AG Manji GA Chabot JA Bates SE. Neoadjuvant treatment for pancreatic cancer. Semin Oncol (2019) 46:19–27. doi: 10.1053/j.seminoncol.2018.12.002 30630600

[16] Mellon EA Hoffe SE Springett GM Frakes JM Strom TJ Hodul PJ. Long-term outcomes of induction chemotherapy and neoadjuvant stereotactic body radiotherapy for borderline resectable and locally advanced pancreatic adenocarcinoma. Acta Oncol (2015) 54:979–85. doi: 10.3109/0284186X.2015.1004367 25734581

[17] Macherla S Laks S Naqash AR Bulumulle A Zervos E Muzaffar M. Emerging role of immune checkpoint blockade in pancreatic cancer. Int J Mol Sci (2018) 19:3505–16. doi: 10.3390/ijms19113505 PMC6274962 30405053

[18] Valero C Lee M Hoen D Zehir A Berger MF Seshan VE. Response rates to anti-PD-1 immunotherapy in microsatellite-stable solid tumors with 10 or more mutations per megabase. JAMA Oncol (2021) 7:739–43. doi: 10.1001/jamaoncol.2020.7684 PMC7893543 33599686

[19] Wei J Lu X Liu Q Li L Liu S Liu F. Case report: Neoadjuvant PD-1 blockade plus concurrent chemoradiotherapy in unresectable locally advanced gastric cancer patients. Front Oncol (2020) 10:554040. doi: 10.3389/fonc.2020.554040 33634011 PMC7901487

[20] Shen D Chen Q Wu J Li J Tao K Jiang Y. The safety and efficacy of neoadjuvant PD-1 inhibitor with chemotherapy for locally advanced esophageal squamous cell carcinoma. J Gastrointest Oncol (2021) 12:1–10. doi: 10.21037/jgo-20-599 33708420 PMC7944149

[21] Duan H Wang T Luo Z Tong L Dong X Zhang Y. Neoadjuvant programmed cell death protein 1 inhibitors combined with chemotherapy in resectable non-small cell lung cancer: An open-label, multicenter, single-arm study. Transl Lung Cancer Res (2021) 10:1020–8. doi: 10.21037/tlcr-21-130 PMC7947385 33718040

[22] Li X Xu C Qiu H Chen D Zhu K Zhang B. A single-arm, multicenter, phase II clinical study of tislelizumab plus albumin-bound paclitaxel/cisplatin as neoadjuvant therapy for borderline resectable esophageal squamous cell carcinoma. Ann Transl Med (2022) 10:263. doi: 10.21037/atm-21-6931 35402596 PMC8987875

[23] Jian D Qian C Wang D Ma Q Wang L Li C. Conversion therapy with tislelizumab for high microsatellite instability, unresectable stage III gastric cancer: A case report. Ann Trans Med (2021) 9:1489–9. doi: 10.21037/atm-21-4295 PMC8506721 34734041

[24] Zhang Q Liu X Wei S Zhang L Tian Y Gao Z. Lenvatinib plus PD-1 inhibitors as first-line treatment in patients with unresectable biliary tract cancer: A single-arm, open-label, phase II study. Front Oncol (2021) 11:751391. doi: 10.3389/fonc.2021.751391 34900698 PMC8651538

[25] Cohen JD Javed AA Thoburn C Wong F Tie J Gibbs P. Combined circulating tumor DNA and protein biomarker-based liquid biopsy for the earlier detection of pancreatic cancers. Proc Natl Acad Sci U.S.A. (2017) 114:10202–7. doi: 10.1073/pnas.1704961114 PMC5617273 28874546

[26] Groot VP Mosier S Javed AA Teinor JA Gemenetzis G Ding D. Circulating tumor DNA as a clinical test in resected pancreatic cancer. Clin Cancer Res (2019) 25:4973–84. doi: 10.1158/1078-0432.CCR-19-0197 PMC7403524 31142500

[27] Lee B Lipton L Cohen J Tie J Javed AA Li L. Circulating tumor DNA as a potential marker of adjuvant chemotherapy benefit following surgery for localized pancreatic cancer. Ann Oncol (2019) 30:1472–8. doi: 10.1093/annonc/mdz200 PMC6771221 31250894

[28] Campos-Carrillo A Weitzel JN Sahoo P Rockne R Mokhnatkin JV Murtaza M. Circulating tumor DNA as an early cancer detection tool. Pharmacol Ther (2020) 207:107458. doi: 10.1016/j.pharmthera.2019.107458 31863816 PMC6957244

[29] Gillen S Schuster T Meyer Zum Buschenfelde C Friess H Kleeff J. Preoperative/neoadjuvant therapy in pancreatic cancer: A systematic review and meta-analysis of response and resection percentages. PloS Med (2010) 7:e1000267. doi: 10.1371/journal.pmed.1000267 20422030 PMC2857873

[30] Heinrich S Pestalozzi BC Schafer M Weber A Bauerfeind P Knuth A. Prospective phase II trial of neoadjuvant chemotherapy with gemcitabine and cisplatin for resectable adenocarcinoma of the pancreatic head. J Clin Oncol (2008) 26:2526–31. doi: 10.1200/JCO.2007.15.5556 18487569

[31] Murphy JE Wo JY Ryan DP Clark JW Jiang W Yeap BY. Total neoadjuvant therapy with FOLFIRINOX in combination with losartan followed by chemoradiotherapy for locally advanced pancreatic cancer: A phase 2 clinical trial. JAMA Oncol (2019) 5:1020–7. doi: 10.1001/jamaoncol.2019.0892 PMC6547247 31145418

[32] Philip PA Lacy J Portales F Sobrero A Pazo-Cid R Manzano Mozo JL. Nab-paclitaxel plus gemcitabine in patients with locally advanced pancreatic cancer (LAPACT): A multicentre, open-label phase 2 study. Lancet Gastroenterol Hepatol (2020) 5:285–94. doi: 10.1016/s2468-1253(19)30327-9 31953079

[33] Cloyd JM Chen HC Wang X Tzeng CD Kim MP Aloia TA. Chemotherapy versus chemoradiation as preoperative therapy for resectable pancreatic ductal adenocarcinoma: A propensity score adjusted analysis. Pancreas (2019) 48:216–22. doi: 10.1097/MPA.0000000000001231 30629022

[34] Gemenetzis G Groot VP Blair AB Laheru DA Zheng L Narang AK. Survival in locally advanced pancreatic cancer after neoadjuvant therapy and surgical resection. Ann Surg (2019) 270:340–7. doi: 10.1097/SLA.0000000000002753 PMC6985003 29596120

[35] Clement-Colmou K Potiron V Pietri M Guillonneau M Jouglar E Chiavassa S. Influence of radiotherapy fractionation schedule on the tumor vascular microenvironment in prostate and lung cancer models. Cancers (Basel) (2020) 12:121–130. doi: 10.3390/cancers12010121 PMC7017121 31906502

[36] Badiyan SN Molitoris JK Chuong MD Regine WF Kaiser A. The role of radiation therapy for pancreatic cancer in the adjuvant and neoadjuvant settings. Surg Oncol Clin N Am (2017) 26:431–53. doi: 10.1016/j.soc.2017.01.012 28576181

[37] Shaib WL Sayegh L Zhang C Belalcazar A Ip A Alese OB. Induction therapy in localized pancreatic cancer. Pancreas (2019) 48:913–9. doi: 10.1097/MPA.0000000000001353 31268982

[38] Huber M Brehm CU Gress TM Buchholz M Alashkar Alhamwe B von Strandmann EP. The immune microenvironment in pancreatic cancer. Int J Mol Sci (2020) 21:7307–39. doi: 10.3390/ijms21197307 PMC7583843 33022971

[39] Li KY Yuan JL Trafton D Wang JX Niu N Yuan CH. Pancreatic ductal adenocarcinoma immune microenvironment and immunotherapy prospects. Chronic Dis Transl Med (2020) 6:6–17. doi: 10.1016/j.cdtm.2020.01.002 32226930 PMC7096327

[40] Ho TTB Nasti A Seki A Komura T Inui H Kozaka T. Combination of gemcitabine and anti-PD-1 antibody enhances the anticancer effect of M1 macrophages and the Th1 response in a murine model of pancreatic cancer liver metastasis. J Immunother Cancer (2020) 8:e001367. doi: 10.1136/jitc-2020-001367 33188035 PMC7668383

[41] Carvalho HA Villar RC. Radiotherapy and immune response: The systemic effects of a local treatment. Clinics (Sao Paulo) (2018) 73:e557s. doi: 10.6061/clinics/2018/e557s 30540123 PMC6257057

[42] Lee YH Yu CF Yang YC Hong JH Chiang CS. Ablative radiotherapy reprograms the tumor microenvironment of a pancreatic tumor in favoring the immune checkpoint blockade therapy. Int J Mol Sci (2021) 22:2091–2103. doi: 10.3390/ijms22042091 PMC7923299 33669885

[43] Fujiwara K Saung MT Jing H Herbst B Zarecki M Muth S. Interrogating the immune-modulating roles of radiation therapy for a rational combination with immune-checkpoint inhibitors in treating pancreatic cancer. J Immunother Cancer (2020) 8:e000351. doi: 10.1136/jitc-2019-000351 32675194 PMC7368549

[44] Ballehaninna UK Chamberlain RS. The clinical utility of serum CA 19-9 in the diagnosis, prognosis and management of pancreatic adenocarcinoma: An evidence based appraisal. J Gastrointest Oncol (2012) 3:105–19. doi: 10.3978/j.issn.2078-6891.2011.021 PMC3397644 22811878

[45] Bettegowda C Sausen M Leary RJ Kinde I Wang Y Agrawal N. Detection of circulating tumor DNA in early- and late-stage human malignancies. Sci Transl Med (2014) 6:224ra24. doi: 10.1126/scitranslmed.3007094 PMC4017867 24553385

[46] Jin Y Chen DL Wang F Yang CP Chen XX You JQ. The predicting role of circulating tumor DNA landscape in gastric cancer patients treated with immune checkpoint inhibitors. Mol Cancer (2020) 19:154. doi: 10.1186/s12943-020-01274-7 33126883 PMC7596978

[47] Goldberg SB Narayan A Kole AJ Decker RH Teysir J Carriero NJ. Early assessment of lung cancer immunotherapy response *via* circulating tumor DNA. Clin Cancer Res (2018) 24:1872–80. doi: 10.1158/1078-0432.CCR-17-1341 PMC5899677 29330207

[48] Zhou J Wang C Lin G Xiao Y Jia W Xiao G. Serial circulating tumor DNA in predicting and monitoring the effect of neoadjuvant chemoradiotherapy in patients with rectal cancer: A prospective multicenter study. Clin Cancer Res (2021) 27:301–10. doi: 10.1158/1078-0432.CCR-20-2299 33046514

